# Cortisol response in children with cancer and fever during chemotherapy: A prospective, observational study using random serum cortisol levels

**DOI:** 10.1002/cam4.5667

**Published:** 2023-02-03

**Authors:** Ann Boekstegers, Heinrich Schmidt, Mathias Kurzay, Tanja Vallée, Eva Jung, Ilja Dubinski, Rebecca Maxwell, Irene Schmid

**Affiliations:** ^1^ Department of Pediatrics Dr. von Hauner Children's Hospital, University Hospital, LMU Munich Munich Germany

**Keywords:** adrenal insufficiency, chemotherapy, cortisol, glucocorticoids, pediatric oncology

## Abstract

**Background:**

Glucocorticoids are crucial components of the treatment of leukemia and lymphoma. High doses can lead to suppression of the hypothalamic–pituitary–adrenal (HPA) axis and be causative for an impaired stress response during infection. This study aims to evaluate the cortisol response in pediatric oncologic patients during febrile episodes.

**Methods:**

Totally, 75 children and adolescents (5 months—18 years) with fever during chemotherapy were consecutively enrolled in this study. In total, 47 patients received glucocorticoids as part of their treatment. Random serum cortisol and adrenocorticotropic hormone (ACTH) were analyzed in every patient. A low cortisol response (LCR) was defined as a cortisol level < 14.6 μg/dL.

**Results:**

In total, 52 (69%) patients had a cortisol level < 14.6 μg/dL during fever. There was no significant difference between patients who received glucocorticoids and those who did not. Significantly lower cortisol levels were measured ≤7 days after last glucocorticoid intake compared to later time points. Nearly all patients treated with dexamethasone or prophylactic posaconazole demonstrated a LCR under stress (fever).

**Conclusion:**

The incidence of an impaired HPA axis in pediatric cancer patients might be underestimated since 69% of the children in our study had a LCR during fever. Intake of dexamethasone, posaconazole and a time period of ≤7 days from the last glucocorticoid intake were additional risk factors for an LCR. However, we could not confirm that patients with a LCR fared worse than patients with a high cortisol response (HCR). Therefore, a different cortisol threshold may be necessary for defining an impaired HPA axis in febrile oncologic patients without concomitant symptoms of AI.

## Graphical abstract

The adrenal gland's response to stress is impaired in most pediatric cancer patients receiving chemotherapy, whether or not they are taking steroids. Intake of dexamethasone and posaconazole and a time period of ≤7 days from the last glucocorticoid intake were additional risk factors for an impaired adrenal response.

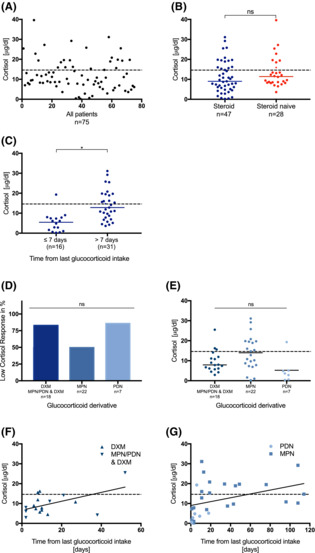

## 1 INTRODUCTION

The hypothalamic–pituitary–adrenal (HPA) axis plays an important role in the stress and immune response to infections. Cortisol has direct anti‐inflammatory effects and inhibits cytokines enhancing the inflammatory cascade.^1^, ^2^ HPA axis suppression and hence reduced adrenal cortisol production remains a cause of morbidity and mortality in children.^3^

Glucocorticoids are an important element in therapeutic regimens of childhood leukemia and lymphoma since they induce apoptosis in lymphoblastic cells.^4^ However, exogenous steroid therapy suppresses corticotropin‐releasing hormone (CRH) and adrenocorticotropic hormone (ACTH) production and thus induces adrenal atrophy.^5^ Several studies have investigated the effects of glucocorticoids on the HPA axis in children with acute lymphoblastic leukemia (ALL). The suppression persists for the first days after the abrupt withdrawal of glucocorticoid therapy. The duration of suppression shows great interpatient variability.^6^, ^7^, ^8^, ^9^, ^10^, ^11^, ^12^, ^13^, ^14^ Supportive therapy, such as opioids or azoles can also suppress the HPA axis.^11^, ^15^, ^16^ Stress in episodes of suppressed HPA axis can have severe consequences.^3^ This is further compounded by the frequent occurrence of chemotherapy induced neutropenia which also increase the susceptibility to infection.^17^, ^18^, ^19^ Published data have shown no evidence of long‐term effects of chemotherapy on the HPA axis to date.^20^, ^21^, ^22^ We would like to further review this topic and hypothesize that polychemotherapy without steroids may also have an impact on the HPA axis. Our hypothesis is that polychemotherapy treatment (with or without steroids) leads to a compromised stress response in the form of inadequate availability of cortisol in this patient group. Serum cortisol levels and ACTH levels were measured in all patients at admission, irrespective of the daytime. We defined a random cortisol level < 14.6 μg/dL as low cortisol response (LCR) in our patients since a cortisol level ≥ 14.6 μg/dL would be expected in patients under stress measured with Cortisol II immunoassay (Roche Diagnostics).^23^, ^24^, ^25^, ^26^

## 2 MATERIALS AND METHODS

### 2.1 Patients

From August 2015 to April 2018, 75 children aged 5 months–18 years (median 4 years) with diagnosed cancer were enrolled in this study. Informed consent was obtained according to the Helsinki declaration and the study was approved by the local medical ethics committee. All patients were undergoing treatment at the Dr. von Hauner Children's Hospital in Munich, Germany, according to the respective study protocol. These children were divided into two groups according to the received pre‐treatment. The steroid group consisted of 47 patients treated for leukemia, lymphoma or Langerhans cell histiocytosis (LCH) (Table 1). The steroid naive group included 28 patients treated for solid tumors or acute myeloid leukemia (Table 2). Eight patients were treated due to a relapse, two in the steroid group, six in the steroid naive group. Patients under 1 month of age or who had had cranial irradiation or with known adrenal‐ or pituitary‐insufficiency were excluded from the study. Test results were not available during the study period and none of the patients received hydrocortisone replacement therapy. Serum for cortisol levels was obtained from the central venous catheter immediately after admission to the hospital and measurement of body temperature. Thereafter antibiotic and antipyretic therapy was started. Fever was defined as body temperature measurement once ≥38.5°C (101.3°F) or twice >38.0°C (100.4°F) at a time interval of ≤1 h.

**TABLE 1:** Diagnosis, therapy and results of the steroid group.

Patient No°	Diagnosis	Time from initial diagnosis (months)	Cumulative equivalent DXM dose†	Time from last glucocorticoid (days)	Cortisol (μg/dL)	ACTH (pg/mL)	Posaconazole prophylaxis
1	NHL	1	90	9	5.7	10.5	No
2	NHL	0	90	9	16.2	25.3	No
3	NHL	1	90	13	4.5	13	No
4	ALL Relapse	0	130	8	11.9	601	Yes
5	NHL	2	160	6	9	13.9	No
6	NHL	2	160	27	10.8	5.1	No
7	HL	2	180	18	5.1	3.4	No
8	ALL	0	210	2	2.9	6.3	No
9	NHL	4	240	10	7.8	15.6	No
10	LCH	4	272	1	0.4	11.4	No
11	NHL	4	280	7	7.4	4.4	No
12	NHL	3	280	10	6.7	11.1	No
13	NHL	1	336	1	0.6	7.8	No
14	LCH	3	384	2	2.8	79.6	No
15	ALL relapse	7	414	8	15.6	31.5	Yes
16	ALL	1	420	1	7.3	43.6	No
17	ALL	1	420	1	1.6	6.8	No
18	ALL	2	420	3	0.2	3.7	No
19	ALL	1	420	3	1	7.9	No
20	ALL	1	420	3	7.8	24.2	No
21	ALL	2	420	11	12.8	23.4	No
22	ALL	2	420	11	16	6.1	No
23	ALL	1	420	11	31.1	6.2	No
24	ALL	2	420	13	25.8	20.4	No
25	ALL	2	420	16	6.9	5.6	No
26	ALL	1	420	19	15.3	16.8	No
27	ALL	1	420	19	20.8	127	No
28	ALL	2	420	34	19.8	5.9	No
29	ALL	2	420	37	8.7	7.2	No
30	ALL	3	420	43	9.7	10	No
31	ALL	3	420	45	18.9	7.1	No
32	ALL	2	420	49	19.7	157	No
33	ALL	4	420	76	19.5	4.8	No
34	NHL	2	420	96	10.8	5.7	No
35	ALL	5	420	108	10.2	3.7	No
36	ALL	5	420	108	29.2	148	No
37	ALL	5	420	114	14.7	21.6	No
38	HL	3	480	6	19.3	6.6	No
39	HL	3	480	9	3.6	8.8	No
40	NHL	8	546	2	6.1	6.1	Yes
41	ALL	6	560	2	6.1	8.5	No
42	ALL	8	560	21	14.1	31.8	No
43	ALL	7	560	38	4.2	4.9	No
44	HL	4	600	4	4.8	5	No
45	ALL	3	620	2	8	16.5	No
46	ALL	10	700	14	11.1	22.7	No
47	ALL	10	700	52	25.5	35.6	No

† Conversion of cumulative MPN dose/m^2^ to equivalent cumulative DXM dose/m^2^ with factor 4, Conversion of cumulative PDN dose/m^2^ to equivalent cumulative DXM dose/m^2^ with factor 5.

Abbreviations: ALL, acute lymphoblastic leukemia; HL, Hodgkin's lymphoma; LCH, Langerhans cell histiocytosis; NHL, Non‐Hodgkin's lymphoma

**TABLE 2:** Diagnosis, therapy and results of the steroid naive group.

Patient No°	Diagnosis	Time from initial diagnosis (months)	Number of chemotherapy cycles	Cortisol (μg/dL)	ACTH (pg/mL)	Posaconazole prophylaxis
1	AML	1	1	8.6	8.9	Yes
2	AML relapse	1	1	8.3	9.3	Yes
3	Ewing's sarcoma	6	6	11.6	22.6	No
4	Ewing's sarcoma	1	2	13.1	32.7	No
5	Ewing's sarcoma	3	3	4.0	25.4	No
6	Ewing's sarcoma	2	3	19.4	13.7	No
7	Osteosarcoma	9	13	8.7	15.7	Yes
8	Osteosarcoma relapse	4	2	9.6	5.8	No
9	Osteosarcoma relapse	6	5	18.5	7.6	No
10	Nephroblastoma	1	5	9	9.9	No
11	Nephroblastoma	0.5	1	8.2	14.2	No
12	Nephroblastoma	3	11	15.9	9.1	No
13	Rhabdomyosarcoma	1	3	12.3	2.7	No
14	Rhabdomyosarcoma	2	6	3.6	14	No
15	Rhabdomyosarcoma	1	6	22.8	44.7	No
16	Hepatoblastoma relapse	18	16	13.4	12.1	No
17	Hepatoblastoma relapse	2	5	13.4	23.5	No
18	Hepatoblastoma	7	7	27.2	21.4	No
19	Hepatoblastoma/HCC	1	2	39.6	308	No
20	Germ cell tumor	6	5	8.5	13.4	No
21	Germ cell tumor	6	4	19.6	26.4	No
22	Neuroblastoma	3	4	8.1	25.2	No
23	Neuroblastoma relapse	2	2	6.5	15.2	Yes
24	Neuroblastoma	3	4	7	6.9	No
25	Neuroblastoma	1	2	12.1	13	No
26	Neuroblastoma	21	14	7.5	18.9	No
27	Astrocytoma	17	22	11.2	27.5	No
28	Medulloblastoma	5	7	9.4	21.3	No

Abbreviations: AML, acute myeloid leukemia; HCC, hepatocellular carcinoma.

### 2.2 Drug assays

Serum cortisol levels and ACTH levels were analyzed in all patients. LCR was defined by a random cortisol level < 14.6 μg/dL (=403 nmoL/L).^23^, ^24^, ^25^ Baseline concentration reference level for ACTH were 10–50 pg/mL (2.2–11 pmoL/L). Cortisol level and ACTH level were measured by electrochemiluminescence with the Cortisol II and Elecsys® ACTH immunoassay (Roche Diagnostics).

### 2.3 Statistics

Continuous data were expressed as median with range values and compared by the Wilcoxon–Mann–Whitney test. The Kruskal–Wallis and Fisher's exact test were used selectively to compare categorical variables where appropriate. In total, six tests for differences between subgroups of patients were applied. Bonferroni correction was applied to adjust for multiple testing. Consequently, *p* values < 0.05/6 = 0.008 were considered statistically significant. Data analyses were performed using GraphPad Prism Version 7.0.

## 3 RESULTS

### 3.1 Clinical data

The 75 patients included in our study demonstrated the common distribution of childhood cancer with the exception of a low rate of brain tumors since cranial irradiation was an exclusion criteria (Data S1).^27^ There was no difference in baseline characteristics between patients receiving glucocorticoids and those who did not (Table 3). The duration of therapy before inclusion in the study was not significantly different, with a median of 2 months in the steroid group and 3 months in the steroid‐naive group. Tables 1 and 2 summarize the diagnosis and pretreatment history of patients with corticosteroids and those without, respectively. We retrospectively verified that the “steroid‐naive” patients also did not receive steroids for other indications such as antiemesis.

**TABLE 3:** Baseline characteristics of the participants.

Characteristic	Treatment with glucocorticoids *n* = 47	Treatment without glucocorticoids *n* = 28	Odds ratio (95% CI) with reference = Treatment without corticosteroids
Age—year			
Median	5	6	
Interquartile range	2–12.5	2–10	
Range	0.4–17	1–17	
Sex			
Male—no. (%)	29 (61.7)	15 (53.6)	1.40 (0.45–2.98)
Female—no. (%)	18 (38.3)	13 (46.4)	
Length of treatment before inclusion (months)			
Median	2	3	
Interquartile range	1–4	1–6	
Range	0.5–10	0.5–18	
Clinical presentation			
Infection focus—no. (%)	13 (27.6)	13 (46.4)	0.44 (0.26–1.87)
Arterial hypotension/Tachycardia—no. (%)	1 (2.1)	4 (14)	0.13 (0.04–3.9)
Laboratory diagnostic			
Neutropenia (<500 neutrophils/μL)—no. (%)	30 (63.8)	19 (67.9)	0.84 (0.34–2.49)
Fever without neutropenia (>500 neutrophils/μL)—no. (%)	17 (36.2)	9 (32.1)	1.20 (0.40–2.91)
C‐reactive protein (CRP) at presentation (>0.5 mg/dL)—no. (%)	29 (61.7)	22 (78.6)	0.44 (0.24–2.05)
Germ proof in blood culture—no.	1 (2.1)	1 (3.5)	1.43 (0.05–13.21)

Abbreviations: CI, confidence interval.

Table 4 summarizes retrospectively collected clinical and laboratory data of patients with cortisol <14.6 μg/dL (52 patients) and ≥14.6 μg/dL (23 patients). The median duration of fever was 2 days in both groups and the median duration of hospitalization was 3 days in patients with LCR and 4 days in those with high cortisol response (HCR). Eleven patients (21%) with cortisol <14.6 μg/dL and seven patients (30%) with cortisol ≥14.6 μg/dL were reported to have malaise at admission. Other symptoms of adrenal insufficiency, such as hypotension, tachycardia, nausea, muscle pain or abdominal pain were rare. Eight patients (15.4%) with cortisol <14.6 μg/dL and three patients (13%) with cortisol ≥14.6 μg/dL presented with electrolyte abnormalities (hyponatremia or hyperkalemia). As blood glucose was not measured in all patients on admission, we cannot assess the occurrence of hypoglycemia as a sign of adrenal insufficiency. About 35% of patients presented with an infective focus, 65% with neutropenia, and 68% showed increased C‐reactive protein levels at admission.

**TABLE 4:** Clinical and laboratory characteristics of patients with a low cortisol response and high cortisol response.

Characteristic	Cortisol <14.6 μg/dL *n* = 52	Cortisol ≥14.6 μg/dL *n* = 23	Odds Ratio (95% CI) With Reference = Cortisol ≥14.6 μg/dL
Clinical presentation			
Symptoms of AI	11 (21.1)	7 (30.4)	0.61 (0.27–2.45)
Nausea/Vomiting/Diarrhea	1 (1.9)	1 (4.3)	
Malaise	11 (21.1)	7 (30.4)	
Muscle/joint pain	0	1	
Abdominal pain	1	0	
Infection focus—no. (%)	18 (34.6)	8 (34.8)	0.99 (0.36–2.79)
Arterial hypotension/Tachycardia—no. (%)	4 (7.7)	1 (4.3)	1.83 (0.27–2.45)
Fever (°C) at presentation			
Median (Interquartile range)	38.6 (38.4–38.9)	38.6 (38.5–38.9)	
Duration of fever (days)			
Median (Interquartile range)	2 (1–3)	2 (1–3.5)	
Length of hospital stay (days)			
Median (Interquartile range)	3 (2–5)	4 (2.5–5)	
Laboratory diagnostic			
Hyponatremia/hyperkalemia—no. (%)	8 (15.4)	3 (13)	1.21 (0.26–4.54)
Neutropenia (<500 neutrophils/μL)—no. (%)	33 (63.5)	16 (69.6)	0.76 (0.31–2.54)
C‐reactive protein (CRP) at presentation (>0.5 mg/dL)—no. (%)	35 (67.3)	16 (69.6)	0.90 (0.33–2.76)
Albumin level <2.5 g/dL	2 (3.8)	0	
Steroid Group (*n* = 47)	*n* = 32	*n* = 15	
ACTH level			
Low (< 10 pg/mL)—no. (%)	17 (53.1)	6 (40)	
Normal (10–50 pg/mL)—no. (%)	13 (40.6)	6 (40)	
High (> 50 pg/mL)—no.(%)	2 (6.3)	3 (20)	
Steroid naive Group (*n* = 28)	*n* = 20	*n* = 8	
ACTH level			
Low (< 10 pg/mL)—no. (%)	6 (30)	2 (25)	
Normal (10–50 pg/mL)—no. (%)	14 (70)	4 (50)	
High (> 50 pg/mL)—no. (%)	0 (0)	2 (25)	

Abbreviations: ACTH, adrenocorticotropic hormone; AI, adrenal insufficiency; CI, confidence interval;

In summary, we cannot confirm that patients with a lower cortisol response fared worse than patients with a higher cortisol response.

Seven of the 75 patients were on antifungal prophylaxis. None of our patients had received opioids at the day of or week prior to admission nor was on long‐term opiate treatment. Two patients showed hypoalbuminemia at admission.

### 3.2 Serum Cortisol levels

**FIGURE 1:**
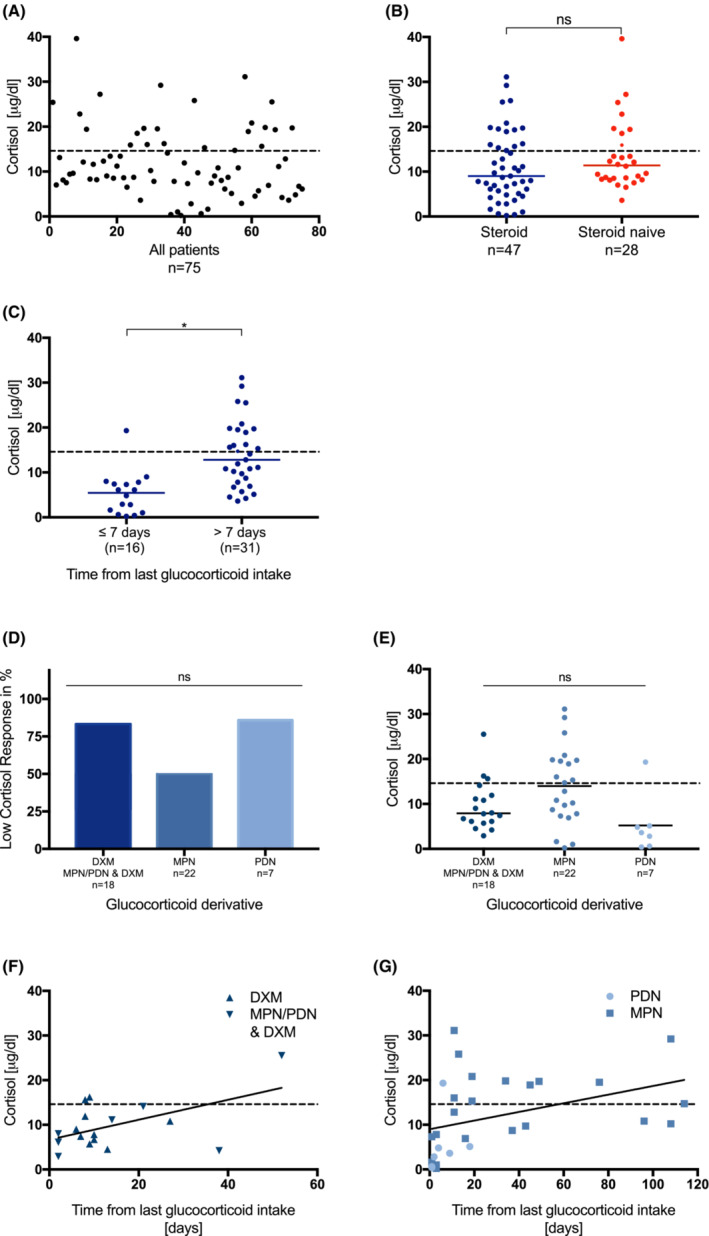
(A) Serum cortisol values in all 75 patients. (B) Serum cortisol values of steroid group and steroid naive group. Wilcoxon–Mann–Whitney test (*p* = 0.1145, U = 513.5). (C) Serum cortisol values with regards to time from last glucocorticoid intake. Wilcoxon–Mann–Whitney test (*p* = <0.0001, U = 74.5). (D) Incidence of LCR with regards to different glucocorticoid derivatives. Kruskal–Wallis test (*p* = 0.0472, chi‐square = 6.106). (E) Serum cortisol values with regards to different glucocorticoid derivatives. Kruskal–Wallis test (*p* = 0.0101, chi‐square = 9.182). (F) Serum cortisol values of patients with DXM or PDN/MPN and DXM plotted against time from last glucocorticoid intake. Spearman correlation (*r* = 0.5427, *p* = 0.02, 95% CI = 0.1016–0.8055). (G) Serum cortisol values of patients with PDN or MPN plotted against time from last glucocorticoid intake. Spearman correlation (*r* = 0.393, *p* = 0.0349, 95% CI = 0.03097–0.6639). *, <0.008, ns, not significant, dashed line = 14.6 μg/dL cortisol.

**FIGURE 2:**
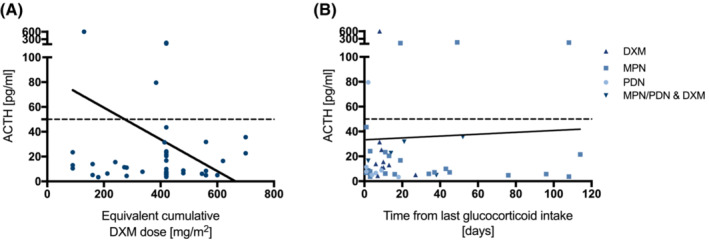
(A) Serum ACTH values plotted against the equivalent cumulative DXM dose. Spearman correlation (*r* = 0.03495, *p* = 0.8156, 95% CI = 0.3267–0.2629). (B) Serum ACTH values plotted against time from the last glucocorticoid intake and marked according to the glucocorticoid derivative. Spearman correlation (*r* = 0.03627, *p* = 0.8087, 95% CI = 0.3279–0.2617).

In total, 52 of 75 (69%) patients had a cortisol level < 14.6 μg/dL (median 9.7 μg/dL; 0.2–39.6 μg/dL) (Figure 1A, Figure 2); 32 of 47 (68%) patients in the steroid group, and 20 of 28 (71%) patients in the steroid naive group presented with a low cortisol level in the febrile state. The median cortisol level under fever was higher (median 11.4 μg/dL; 3.6–39.6 μg/dL) in the naive group than in the steroid group (median 9.0 μg/dL; 0.2–31.1 μg/dL). The comparison of absolute cortisol levels in both groups showed no statistically significant result (*p* = 0.1145) (Figure 1B).

Seven out of 75 patients were on posaconazole as antifungal prophylaxis and six of them showed low cortisol responses (median 8.5 μg/dL; 6.1–11.9 μg/dL). Two of them received glucocorticoids, four of them did not.

The analysis of cortisol levels with regard to the time from the last glucocorticoid intake revealed significantly lower levels of cortisol within 7 days of last intake compared to later time points (*p* ≤ 0.0001) (Figure 1C). The time from the last glucocorticoid intake correlated positively with the cortisol value (*r* = 0.6165, *p* ≤ 0.0001).

Patients with glucocorticoids in their treatment protocol received either dexamethasone (DXM), methylprednisolone (MPN), prednisolone (PDN), or a combination of the latter over a certain period with or without tapering (Data S2). Further analysis of the different derivatives revealed no difference in the incidence of LCR (*p* = 0.0472) or in absolute values of cortisol (*p* = 0.01) (Figure 1D,E). The median cortisol level under fever was highest in MPN patients (median 13.8 μg/dL; 2.9–25.5 μg/dL) with a median time from last MPN intake of 19 days and 50% incidence of LCR (Data S5). About 83% children receiving DXM, MPN, or PDN followed by DXM had random cortisol levels <14.6 μg/dL. The median time from the last DXM intake was 9 days, the median cortisol level 7.9 μg/dL (2.9–25.5 μg/dL) (Data S3). In total, 86% patients receiving PDN had a lower cortisol response; the median time from the last PDN intake was 4 days, the median cortisol level 3.6 μg/dL (Data S4).

### 3.3 ACTH levels

In the steroid group, 30 of 32 patients with low cortisol levels had low or normal ACTH level. Two patients had low cortisol but high ACTH (Table 4).

**FIGURE 3:**
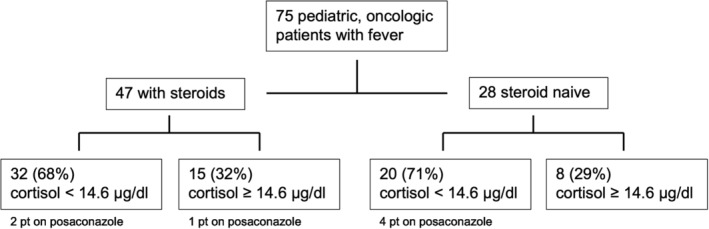
Flow chart of patient cohort. pt, patients.

Table 1 summarizes the diagnosis, time from initial diagnosis, cumulative equivalent DXM dose, the time since the last glucocorticoid intake, and the patients' respective cortisol, and ACTH levels. To better compare the effect of cumulative steroid doses, we converted the doses to equivalent cumulative dexamethasone doses (Data S6). Neither the cumulative equivalent DXM dose nor time from last steroid intake correlated with ACTH levels (Figure 3A,B). Likewise, the equivalent cumulative DXM dose did not correlate with cortisol levels (*r* = 0.1801, *p* = 0.2258). The table illustrates that inter‐individual variability in stress response is large. This is particularly striking when comparing patients who received the same equivalent cumulative dose of DXM at the same interval from the last dose (Pat.No°1 and 2, 16 and 17, 18–20, 21–23, 26 and 27, 35 and 36).

In the steroid naive group, all 20 patients with low cortisol levels had low or normal ACTH levels. Four of these twenty patients were treated prophylactically with posaconazole. None of the patients without steroids had high ACTH level despite a low cortisol level (Table 4).

## 4 DISCUSSION

This is the first study to demonstrate that 69% of children and adolescents with cancer who were receiving chemotherapy had a low cortisol response to fever, irrespective of whether glucocorticoid treatment had been given before or not. Previous studies have shown that the adrenal recovery after corticosteroid treatment can take several weeks in pediatric ALL patients. However, prolonged suppression lasting longer than several months has been reported.^6^, ^7^, ^8^, ^9^, ^10^, ^11^, ^12^, ^13^, ^14^ Therefore, some authors recommend considering steroid replacement therapy during periods of serious stress to reduce the risk of complications.^6^, ^7^, ^10^, ^12^ None of these studies explicitly assessed the adrenal function in a cohort under stress (fever).^14^ In addition, there are no data on adrenal function in children with fever receiving chemotherapy without glucocorticoids in their treatment regimen.

Our study addresses important and clinically relevant questions in this patient cohort. All our patients were admitted to the hospital due to fever, which is generally regarded as a sufficient stimulus for the HPA axis to produce cortisol. Hence, we decided to measure random cortisol and ACTH at admission, irrespective of the daytime. We decided against additional dynamic tests since repeated blood draws from central venous lines are an infection hazard and a delayed antibiotic therapy would not have been justifiable in this high‐risk patient cohort.

We defined a random cortisol level < 14.6 μg/dL as LCR in our patients. Previous studies have shown that cortisol level ≥ 18 μg/dL would be expected in patients under stress. This is also the cut‐off value in the low‐dose corticotropin stimulation test.^23^, ^24^, ^25^ It is important to note however, that older studies measured cortisol levels with polyclonal antibody assays that are less accurate compared to newer, monoclonal antibody assays which show less cross‐reactivity to endogenous steroids.^28^, ^29^ Recent work comparing the cortisol II assay (Roche Diagnostics) used in our study with older assays suggests a lower cut‐off value of 14.6 μg/dL as equivalent to the 18 μg/dL used in older assays.^26^ We therefore adopted this approach to avoid overdiagnosis of adrenal insufficiency (AI).

Various studies have shown that adrenal suppression is always present immediately after cessation of glucocorticoid therapy and may persist up to several months.^7^, ^11^, ^14^ In our study, the cortisol values under stress correlated with the time from the last intake, thus we could confirm this trend. There was a significant difference in the cortisol values between patients with ≤7 days and >7 days since last intake in our study. Therefore, we assume that patients are at higher risk during the earlier timeframe. In contrast, the cumulative corticosteroid dose did not correlate with the cortisol level.

Previous studies have compared the occurrence of AI between PDN and DXM in pediatric oncology patients. Two randomized controlled studies found no difference and one observational study demonstrated earlier recovery in children receiving PDN.^8^, ^9^, ^13^, ^30^ Regarding the occurrence of LCR, we could not observe a significant difference between the various steroid derivatives. Nevertheless, we noticed a high incidence (83%) of LCR in patients receiving DXM or MPN/PDN followed by DXM. Those patients had a median time from the last DXM intake of 9 days, reflecting the strong and long biological effect of DXM. We assume that the high incidence of LCR in the PDN group (86%) is mostly due to the median interval of only 4 days to the last corticosteroid use.

As would be expected for suppressed adrenal glands in our patients with glucocorticoid therapy, 30 of 47 patients (64%) had low or normal ACTH as well as low cortisol levels under stress. Two patients had low cortisol but high ACTH level, as expected in primary AI or recovery of adrenal function after long‐term suppression. We could not see any correlation between cumulative glucocorticoid dose or interval from last glucocorticoid intake and ACTH levels. However, we observed that the interindividual variability of the stress response is large, especially when comparing patients who received the same equivalent cumulative steroid dose and had the same interval from the last dose.

About 20 of 28 (71%) patients in the steroid naive group had low or normal ACTH level despite low cortisol level, consistent with secondary AI. In four of these patients' prophylactic antifungal treatment with posaconazole might be causative, as discussed below. As none of these patients were treated with opioids at admission or during the week before, this could be excluded as a cause. Besides the explanation that fever might not have been enough stimulus for the HPA axis to elicit a higher ACTH response, chronic inflammation from the cancer and/or polychemotherapy could also be causative for the low cortisol response in patients that were steroid naive. It is suggested that patients with chronic inflammatory disease may have chronically elevated cortisol levels but lose their HPA axis reactivity to more acute stressors.^31^

None of the patients without steroids had high ACTH level despite a low cortisol level, as expected in primary AI. To our knowledge, previous studies did not report a contribution of chemotherapy to adrenal dysfunction except for Mitotane used for adrenal carcinoma.^20^, ^32^, ^33^ It is important to further validate our observation in patients without glucocorticoids in their treatment schedule since it would be hazardous to oversee this potentially fatal complication in this patient cohort.

We retrospectively recorded how patients with a cortisol <14.6 μg/dL fared as compared to those with a higher value. Since our study was noninterventional and none of our patient's received hydrocortisone after admission, the clinical course was not confounded. In conclusion, we cannot confirm that patients with a low cortisol response fared worse than patients without. There was no significant difference in clinical presentation, duration of fever and hospital stay duration. There are limitations to these results. Data were collected retrospectively from admission findings and were not systematically collected by the admitting physician. We cannot exclude the possibility that vital signs documented in the patient's chart were measured after the administration of antipyretic therapy. Concomitant symptoms of adrenal insufficiency, such as hypoglycemia, were not systematically recorded. In addition, signs and symptoms of AI may have been so subtle that they were not recognized.

Another important observation of our study is the high incidence of low cortisol response in patients under posaconazole prophylaxis. Fluconazole therapy was evaluated as a risk factor for persistence of AI in two cohort studies in children treated for ALL, but no data exist on posaconazole and adrenal suppression in children.^11^, ^13^ Two case reports in adults report about posaconazole‐related AI, one in a patient with diabetes mellitus and another in a patient with chronic myelomonocytic leukemia.^34^, ^35^ A possible explanation for a prolonged suppression of the adrenal gland might be the inhibition of the cytochrome P450‐dependent CYP3A4 that can lead to a decreased hepatic metabolism of synthetic glucocorticoid.^36^

Hypoalbuminemia caused by malnutrition could also cause subnormal total cortisol values under stress in our patients.^37^, ^38^ Since only two of our 75 patients had hypoalbuminemia, we do not think that this affected the result. Additionally, corticosteroid‐binding globulin (CBG) binding to cortisol is reported to be temperature responsive. Increasing body temperature decreases the CBG concentrations thereby increasing free cortisol levels.^39^ Our total cortisol level might therefore underestimate the HPA axis reserve in our patients. However, recent studies demonstrated that serum total cortisol levels were strongly correlated with free cortisol levels measured in blood or saliva in children with septic shock.^25^, ^40^, ^41^, ^42^ Another limitation is that free cortisol assays are not readily available in every clinic. We therefore decided to measure serum total cortisol levels.

Two patients on PDN and two patients on MPN received the last corticosteroid dose the day before admission with fever. The biologic half‐life of PDN and MPN is reported to be 18–36 h. The Cortisol II assay (Roche diagnostics) used in our study states a cross‐reactivity for these two derivatives. Therefore, we cannot exclude a falsely high result in those patients. Since these four patients had low cortisol levels (range 0.4–7.3 μg/dL), we do not think this affected the results.

Several explanations should be considered for the presented findings. First, as no baseline HPA axis evaluation was performed in the study, an AI could already have been present at the time of the cancer diagnosis. Yet, no reports of such an adrenal dysfunction at the time of oncological diagnosis have been found in review of the literature.^7^, ^9^, ^12^ Second, we did not have a control group of healthy children with fever. The study by Nickels et al. showed a random cortisol level of 29.7 ± 1.5 μg/dL in 76 healthy children with fever without hypotension. This cortisol level was determined at admission with fever, independent of the daytime and was therefore comparable to our cohort.^43^ Other studies analyzing morning cortisol levels in febrile, healthy children also showed levels >18 μg/dL.^44^, ^45^ Therefore, we hypothesized that fever should result in an adequate adrenal response in our cohort even in the absence of hypotension. However, there might be interindividual differences in the extent of stress associated with febrile illness. Furthermore, it might have been advantageous to include a control group using the same cortisol assay.

Third, serum cortisol was obtained only once on presentation with fever so that the duration and height of cortisol response with time was not elucidated. Cortisol levels are known to rise abruptly with onset of fever and then remain at a plateau before dropping to normal values shortly after initiation of appropriate therapy.^46^ Determination of serum cortisol at presentation with fever should represent the described plateau phase and therefore accurately reflect the magnitude of adrenal response to stress.

Only a few studies have investigated stress as a risk factor for AI in children with cancer and these studies were in patients with glucocorticoids in the therapy regimes. Kuperman et Al. performed weekly low‐dose ACTH tests in 29 ALL patients until 8 weeks after cessation of glucocorticoid treatment. Seven of twenty five episodes of infection or stress occurred in patients with insufficient cortisol levels. The authors found no correlation between the presence of stress and the response to the low‐dose ACTH test. This study reported a remarkably low frequency of AI under stress. However, the patient cohort size was small, there was no information on the time period from the last glucocorticoid intake at the time of fever and some patients had more than one stress episode.^8^ Mahachoklertwattana et al. reported that four children with ALL readmitted between 2 and 4 weeks after completing induction therapy for fever and neutropenia had inappropriately low morning cortisol levels (<15 μg/dL).^10^ In both studies, adrenal function was not analyzed at admission before initiation of antipyretic/antibiotic therapy. This may have influenced the results.

## 5 CONCLUSION

Taking all our results into account, we demonstrated that pediatric cancer patients often show a low cortisol response during fever. Both glucocorticoid treatment and multi‐agent chemotherapy appear to suppress the HPA axis. However, we did not see in the retrospective analysis that patients with a low adrenocortical response fared worse than patients with a higher response. Therefore, a different cortisol threshold may be necessary for defining adrenal insufficiency in febrile oncologic patients, or the current definition should be used only in the presence of concurrent clinical signs of AI, such as hypotension. Special attention should be given to patients who have just finished glucocorticoid treatment, have received DXM or are under treatment with posaconazole. Future studies should examine random cortisol levels as well as ACTH stimulation tests in febrile pediatric oncologic patients to assess the adrenal function more accurately. In addition, clinical symptoms of adrenal insufficiency should be systematically collected to determine the clinical impact.

## AUTHOR CONTRIBUTIONS

**Ann Boekstegers:** Conceptualization (equal); formal analysis (lead); investigation (lead); project administration (lead); writing – original draft (lead); writing – review and editing (lead). **Heinrich Schmidt:** Conceptualization (equal); writing – original draft (supporting); writing – review and editing (supporting). **Mathias Kurzay:** Investigation (supporting); writing – original draft (supporting); writing – review and editing (supporting). **Tanja Vallée:** Investigation (supporting); writing – original draft (supporting); writing – review and editing (supporting). **Eva Jung:** Investigation (supporting); writing – original draft (supporting); writing – review and editing (supporting). **Ilja Dubinski:** Investigation (supporting); writing – original draft (supporting); writing – review and editing (supporting). **Rebecca Maxwell:** Writing – original draft (supporting); writing – review and editing (supporting). **Irene Schmid:** Conceptualization (lead); formal analysis (supporting); project administration (supporting); writing – original draft (supporting); writing – review and editing (supporting).

## FUNDING INFORMATION

This research received no specific grant from any funding agency in the public, commercial, or not‐for‐profit sectors.

## CONFLICT OF INTEREST STATEMENT

The authors declare that they have no known competing financial interests or personal relationships that could have appeared to influence the work reported in this paper.

## ETHICS APPROVAL STATEMENT

Informed consent was obtained according to the Helsinki declaration and the study was approved by the local medical ethics committee.

## Supporting information


Appendix S1.

## Data Availability

The data that support the findings of this study are available on request from the corresponding author. The data are not publicly available due to privacy or ethical restrictions.
