# Supplementary material for: Cortisol response in children with cancer and fever during chemotherapy: A prospective, observational study using random serum cortisol levels
Source: Cancer Med. 2023 Feb 3;12(8):9247–59. doi: 10.1002/cam4.5667 (PMC10166925; doi:10.1002/cam4.5667)
Supplement: Supplementary file 1 — Appendix S1. [file CAM4-12-9247-s001.docx]

Supplements


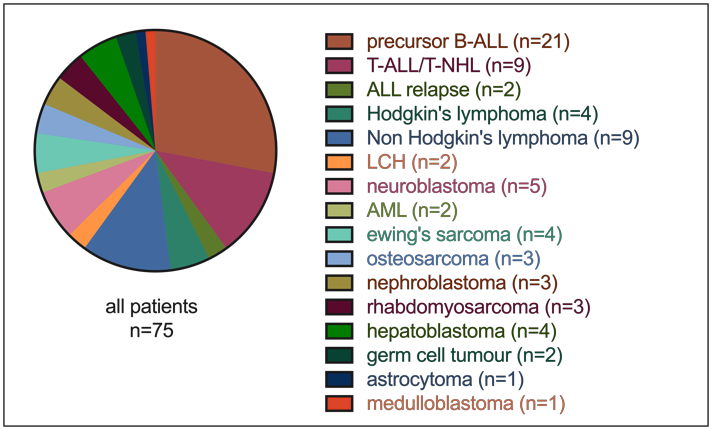


Supplement 1: Distribution of oncological diagnoses in our patient cohort

| **Disease** | **Age** | **Study** | **Protocol phase** | **Dose + Duration**  (orally) | **Tapering** | **Patients Treated** |
| --- | --- | --- | --- | --- | --- | --- |
| precursor B-ALL + T-ALL | ≥ 1 –  ≤ 18 years | COALL 08-09 | Prophase and Induction | MPN 60 mg/m^2^ for 28d | No | n=22 |
|  |  |  | Reinduction | DXM 10 mg/m^2^ for 14d  (1 or 2 cycles depending on risk group) | No | n=5 |
|  |  |  | HR1 | DXM 20 mg/m^2^ for 5d | No | n=1 |
| Precursor B-ALL + T-ALL | ≥ 1 –  ≤ 18 years | AEIOP BFM ALL 2009 | Prephase, IA, IA’, AI-CPM | PDN 60 mg/m^2^ for 28d | Yes | n=1 |
|  |  |  | IA-Dexa, IIA, II Asp+ | DXM 10 mg/m^2^ for 21d | Yes |  |
|  |  |  | HR-1’, HR-2’, HR3’ | DXM 20 mg/m^2^ for 5d | No |  |
|  |  |  | III | DXM 10 mg/m^2^ for 14d | Yes |  |
| Precursor-B-ALL | ≤ 1 year | ALL-Interfant | Induction | PDN 60 mg/m^2^ d1-d7,  DXM 6 mg/m^2^ d8-28 | Yes | n=1 |
|  |  |  | OCTADAD | DXM 6 mg/m^2^ for 15 days | Yes |  |
| ALL relapse | ≤ 18 years | ALL-REZ-BFM | Prephase | DXM 6 mg/m^2^ for 5d | No |  |
|  |  |  | F1/F2/R1/R2 | DXM 20 mg/m^2^ for 5d | No | n=2 |
|  |  |  | II-IDA | DXM 6mg/m^2^ orally for 14d | Yes |  |
| Mature B-NHL/B-AL | < 18 years | NHL-BFM-Registry 2012 | Prephase | DXM 5 mg/m^2^ d1+d2,  DXM 10 mg/m^2^ d3-d5 | No |  |
|  |  |  | A^4^, AA^24^, B^4^, BB^24^ | DXM 10mg/m^2^ for 5 d | No | n=3 |
|  |  |  | AAZ1, AAZ2, BBZ1, BBZ2 | DXM 10 mg/m^2^ for 6 d | No | n=3 |
| Lymphoblastic Lymphoma | < 18 years | NHL-BFM Registry 2012 | Prephase/Induction | PDN 60 mg/m^2^ for 7+21 d | Yes |  |
|  |  |  | Reinduction Protocol II | DXM 10 mg/m^2^ for 21 d | Yes | n=1 |
| ALCL | < 18 years | NHL-BFM Registry 2012 | Prephase | DXM 5 mg/m^2^ d1+d2,  DXM 10 mg/m^2^ d3-d5 | No |  |
|  |  |  | AM, BM | DXM 10mg/m2 for 5d | No | n=2 |
| Hodgkin’s lymphoma | < 18 years | EuroNet-PHL-C1 | OEPA | PDN 60 mg/m^2^ for 15 d | No | n=1 |
|  |  |  | COPDAC, DECOPDAC | PDN 40 mg/m^2^ for 15 d | No | n=3 |
| LCH | < 18 years | LCH-III | Initial treatment course 1+2 | PDN 40 mg/m^2^ for 28 d | Yes | n=1 |
|  |  |  | Continuation treatment | PDN 40 mg/m^2^ for 5 d | No | n=1 |

Supplement 2: Overview of leukaemia, lymphoma and LCH study protocols

| **Disease** | **Patients treated** | **Cumulative Dose of PDN at study entry**  **[mg/m^2^]** | **Cumulative Dose of MPN at study entry [mg/m^2^]** | **Cumulative Dose of DXM at study entry [mg/m^2^]** | **Time from last glucocorticoid [days]** | **Cortisol value under stress [µg/dl]** | **Low Cortisol Response – no. (%)** |  |
| --- | --- | --- | --- | --- | --- | --- | --- | --- |
| ALL relapse | n=2 |  |  | 414  130 | 8  8 | 15.6  11.9 | 1/2 |  |
| Mature aggressive B cell lymphoma & Burkitt’s leukaemia | n=6 |  |  | 90  240  160  160  280  280 | 9  10  6  27  7  10 | 5.7  7.8  9.0.  10.8  7.4  6.7 | 6/6 |  |
| ALCL | n=2 |  |  | 90  90 | 13  9 | 4.5  16.2 | 1/2 |  |
|  |  |  |  | Median  160 | Median  9.5 | Median  8.4 | Total  8/10 (80) |  |
| Precursor-B-ALL  Lymphoblastic Lymphoma | n=1  n=1 | 420  1680 |  | 126  210 | 2  2 | 2.9  6.1 | 1/1  1/1 |  |
|  |  | Median  1050 |  | Median  168 | Median  2 | Median  4.5 | Total  2/2 (100) |  |
| precursor B-ALL + T-ALL | n=6 |  | 1680  1680  1680  1680  1680  1680 | 140  140  140  280  280  200 | 2  21  38  14  52  2 | 6.1  14.1  4.2  11.1  25.5  8.0 | 5/6 |  |
|  |  |  | Median  1680 | Median  170 | Median  17.5 | Median  9.55 | Total  5/6 (83.3) |  |

Supplement 3: Dexamethasone Group

| **Disease** | **Patients treated** | **Cumulative Dose of PDN at study entry**  **[mg/m^2^]** | **Cumulative Dose DXM at study entry [mg/m^2^]** | **Time from last Glucocorticoid [days]** | **Cortisol value under stress**  **[µg/dl]** | **Low Cortisol Response – no. (%)** |
| --- | --- | --- | --- | --- | --- | --- |
| Precursor B-ALL + T-ALL | n=1 | 1680 |  | 1 | 0.6 | 1/1 |
| LCH | n=2 | 1320  1920 |  | 1  2 | 0.4  2.8 | 2/2 |
| Hodgkin’s lymphoma | n=4 | 900  2400  2400  3000 |  | 18  6  9  4 | 5.1  19.3  3.6  4.8 | 3/4 |
|  |  |  |  |  |  |  |
|  |  | Median  1920 |  | Median  4 | Median  3.6 | Total  6/7 (85.7) |
|  |  |  |  |  |  |  |

Supplement 4: Prednisone Group

| **Disease** | **Patients treated** | **Cumulative Dose of MPN at study entry [mg/m^2^]** | **Cumulative Dose DXM at study entry [mg/m^2^]** | **Time from last Glucocorticoid**  **[days]** | **Cortisol value under stress**  **[µg/dl]** | **Low Cortisol Response – no. (%)** |  |
| --- | --- | --- | --- | --- | --- | --- | --- |
| precursor B-ALL + T-ALL | n=22 | 1680  1680  1680  1680  1680  1680  1680  1680  1680  1680  1680  1680  1680  1680  1680  1680  1680  1680  1680  1680  1680  1680 |  | 1  1  3  3  3  11  11  11  13  16  19  19  34  37  43  45  49  76  96  108  108  114 | 7.3  1.6  1.0  0.2  7.8  12.8  16  31.1  25.8  6.9  15.3  20.8  19.8  8.7  9.7  18.9  19.7  19.5  10.8  10.2  29.2  14.7 | 11/22 |  |
|  |  | Median  1680 |  | Median  19 | Median  13.75 | Total  11/22 (50) | |
|  |  |  |  |  |  |  | |

Supplement 5: Methylprednisolone Group

| **Patient No°** | **Diagnosis** | | **Time from initial diagnosis [months]** | **Cumulative Dose at study entry [mg/m2]**  **MPN dose/**  **equivalent DXM dose** | **PDN dose/**  **equivalent DXM dose** | **DXM dose** | **Cumulative equivalent DXM dose*** | **Time from last glucocorticoid**  **[days]** | **Cortisol**  **[µg/dl]** | **ACTH**  **[pg/ml]** | **Posaconazole prophylaxis** | |
| --- | --- | --- | --- | --- | --- | --- | --- | --- | --- | --- | --- | --- |
| 1 | NHL | 1 | |  |  | 90 | 90 | 9 | 5.7 | 10.5 | | No |
| 2 | NHL | 0 | |  |  | 90 | 90 | 9 | 16.2 | 25,3 | | No |
| 3 | NHL | 1 | |  |  | 90 | 90 | 13 | 4.5 | 13 | | No |
| 4 | ALL Relapse | 0 | |  |  | 130 | 130 | 8 | 11.9 | 601 | | Yes |
| 5 | NHL | 2 | |  |  | 160 | 160 | 6 | 9 | 13.9 | | No |
| 6 | NHL | 2 | |  |  | 160 | 160 | 27 | 10.8 | 5.1 | | No |
| 7 | HL | 2 | |  | 900/180 |  | 180 | 18 | 5.1 | 3.4 | | No |
| 8 | ALL | 0 | |  | 420/84 | 126 | 210 | 2 | 2.9 | 6.3 | | No |
| 9 | NHL | 4 | |  |  | 240 | 240 | 10 | 7.8 | 15.6 | | No |
| 10 | LCH | 4 | |  | 1360/272 |  | 272 | 1 | 0.4 | 11.4 | | No |
| 11 | NHL | 4 | |  |  | 280 | 280 | 7 | 7.4 | 4.4 | | No |
| 12 | NHL | 3 | |  |  | 280 | 280 | 10 | 6.7 | 11.1 | | No |
| 13 | NHL | 1 | |  | 1680/336 |  | 336 | 1 | 0.6 | 7.8 | | No |
| 14 | LCH | 3 | |  | 1920/384 |  | 384 | 2 | 2.8 | 79.6 | | No |
| 15 | ALL Relapse | 7 | |  |  | 414 | 414 | 8 | 15.6 | 31.5 | | Yes |
| 16 | ALL | 1 | | 1680/420 |  |  | 420 | 1 | 7.3 | 43.6 | | No |
| 17 | ALL | 1 | | 1680/420 |  |  | 420 | 1 | 1.6 | 6.8 | | No |
| 18 | ALL | 2 | | 1680/420 |  |  | 420 | 3 | 0.2 | 3.7 | | No |
| 19 | ALL | 1 | | 1680/420 |  |  | 420 | 3 | 1 | 7.9 | | No |
| 20 | ALL | 1 | | 1680/420 |  |  | 420 | 3 | 7.8 | 24.2 | | No |
| 21 | ALL | 2 | | 1680/420 |  |  | 420 | 11 | 12.8 | 23.4 | | No |
| 22 | ALL | 2 | | 1680/420 |  |  | 420 | 11 | 16 | 6.1 | | No |
| 23 | ALL | 1 | | 1680/420 |  |  | 420 | 11 | 31.1 | 6.2 | | No |
| 24 | ALL | 2 | | 1680/420 |  |  | 420 | 13 | 25.8 | 20.4 | | No |
| 25 | ALL | 2 | | 1680/420 |  |  | 420 | 16 | 6.9 | 5.6 | | No |
| 26 | ALL | 1 | | 1680/420 |  |  | 420 | 19 | 15.3 | 16.8 | | No |
| 27 | ALL | 1 | | 1680/420 |  |  | 420 | 19 | 20.8 | 127 | | No |
| 28 | ALL | 2 | | 1680/420 |  |  | 420 | 34 | 19.8 | 5.9 | | No |
| 29 | ALL | 2 | | 1680/420 |  |  | 420 | 37 | 8.7 | 7.2 | | No |
| 30 | ALL | 3 | | 1680/420 |  |  | 420 | 43 | 9.7 | 10 | | No |
| 31 | ALL | 3 | | 1680/420 |  |  | 420 | 45 | 18.9 | 7.1 | | No |
| 32 | ALL | 2 | | 1680/420 |  |  | 420 | 49 | 19.7 | 157 | | No |
| 33 | ALL | 4 | | 1680/420 |  |  | 420 | 76 | 19.5 | 4.8 | | No |
| 34 | NHL | 2 | | 1680/420 |  |  | 420 | 96 | 10.8 | 5.7 | | No |
| 35 | ALL | 5 | | 1680/420 |  |  | 420 | 108 | 10.2 | 3.7 | | No |
| 36 | ALL | 5 | | 1680/420 |  |  | 420 | 108 | 29.2 | 148 | | No |
| 37 | ALL | 5 | | 1680/420 |  |  | 420 | 114 | 14.7 | 21.6 | | No |
| 38 | HL | 3 | |  | 2400/480 |  | 480 | 6 | 19.3 | 6.6 | | No |
| 39 | HL | 3 | |  | 2400/480 |  | 480 | 9 | 3.6 | 8.8 | | No |
| 40 | NHL | 8 | |  | 1680/336 | 210 | 546 | 2 | 6.1 | 6.1 | | Yes |
| 41 | ALL | 6 | | 1680/420 |  | 140 | 560 | 2 | 6.1 | 8.5 | | No |
| 42 | ALL | 8 | | 1680/420 |  | 140 | 560 | 21 | 14.1 | 31.8 | | No |
| 43 | ALL | 7 | | 1680/420 |  | 140 | 560 | 38 | 4.2 | 4.9 | | No |
| 44 | HL | 4 | |  | 3000/600 |  | 600 | 4 | 4.8 | 5 | | No |
| 45 | ALL | 3 | | 1680/420 |  | 200 | 620 | 2 | 8 | 16.5 | | No |
| 46 | ALL | 10 | | 1680/420 |  | 280 | 700 | 14 | 11.1 | 22.7 | | No |
| 47 | ALL | 10 | | 1680/420 |  | 280 | 700 | 52 | 25.5 | 35.6 | | No |

Supp.6: Steroid group: Data on diagnosis, time from initial diagnosis, cumulative glucocorticoid dose, time from last glucocorticoid intake, Cortisol & ACTH levels, posaconazole prophylaxis. *Conversion of cumulative MPN dose/m^2^ to equivalent cumulative DXM dose/m^2^ with factor 4, Conversion of cumulative PDN dose/m^2^ to equivalent cumulative DXM dose/m^2^ with factor 5.
